# In Silico Molecular Docking and Simulation Studies of Protein HBx Involved in the Pathogenesis of Hepatitis B Virus-HBV

**DOI:** 10.3390/molecules27051513

**Published:** 2022-02-23

**Authors:** Ibrahim Ahmed Shaikh, Uday M. Muddapur, Krithika C, Shrikanth Badiger, Madhura Kulkarni, Mater H. Mahnashi, Saleh A. Alshamrani, Mohammed A. Huneif, Sunil S. More, Aejaz Abdullatif Khan, S. M. Shakeel Iqubal

**Affiliations:** 1 Department of Pharmacology, College of Pharmacy, Najran University, Najran 66462, Saudi Arabia; i.ibrahimshaikh09@gmail.com; 2 Department of Biotechnology, KLE Technological University, BVB Campus, Hubballi 580031, Karnataka, India; krithika.6500@gmail.com (K.C.); shreebadiger2000@gmail.com (S.B.); madhurakulkarni0111@gmail.com (M.K.); 3 Department of Pharmaceutical Chemistry, College of Pharmacy, Najran University, Najran 66462, Saudi Arabia; matermaha@gmail.com; 4 Department of Clinical Laboratory Sciences, College of Applied Medical Sciences, Najran University, Najran 66462, Saudi Arabia; saalshamrani@nu.edu.sa; 5 Department of Pediatrics, College of Medicine, Najran University, Najran 66462, Saudi Arabia; maalhuneif@nu.edu.sa; 6 School of Basic and Applied Sciences, Dayananda Sagar University, Bangalore 560078, Karnataka, India; sunilsmorey@gmail.com; 7 Department of General Science, Ibn Sina National College for Medical Studies, Jeddah 21418, Saudi Arabia; aeju_kh@yahoo.com (A.A.K.); shakeeliqubal@gmail.com (S.M.S.I.)

**Keywords:** HBx protein, hepatitis B virus, iGEMDOCK, molecular docking, binding energy, ADMET, MD simulation

## Abstract

Current drug discovery involves finding leading drug candidates for further development. New scientific approaches include molecular docking, ADMET studies, and molecular dynamic simulation to determine targets and lead compounds. Hepatitis B is a disease of concern that is a life-threatening liver infection. The protein considered for the study was HBx. The hepatitis B X-interacting protein crystal structure was obtained from the PDB database (PDB ID-3MSH). Twenty ligands were chosen from the PubChem database for further in silico studies. The present study focused on in silico molecular docking studies using iGEMDOCK. The triethylene glycol monoethyl ether derivative showed an optimum binding affinity with the molecular target HBx, with a high negative affinity binding energy of −59.02 kcal/mol. Lipinski’s rule of five, Veber, and Ghose were followed in subsequent ADMET studies. Molecular dynamic simulation was performed to confirm the docking studies and to analyze the stability of the structure. In these respects, the triethylene glycol monoethyl ether derivative may be a promising molecule to prepare future hepatitis B drug candidates. Substantial research effort to find a promising drug for hepatitis B is warranted in the future.

## 1. Introduction

HBV, which belongs to the Hepadnaviridae group, has a small double-stranded circular-DNA genome that is relaxed and converted to covalently closed circular DNA (cccDNA) in the nuclei of infected hepatocytes [1]. Of the four mRNAs generated from cccDNA by the host RNA polymerase 2, the 0.7 kb mRNA encodes the HBV X protein [1]. It has fascinating properties because it is required for HBV infection in the human liver that expresses the 17-kD HBx protein [2]. However, the exact functions of HBx are not entirely understood in the virus lifecycle. An infected person spreads hepatitis B through blood, sperm, or other body fluids to someone who is not infected. The transmission can occur through sexual contact, needle sharing, syringe sharing, or from mother-to-baby [3]. Chronic hepatitis B virus infection, which accounts for 55% of liver cancer cases globally, has been linked to liver carcinogenesis [4]. In the ranking of the most common cancers worldwide, hepatocellular carcinoma (HCC) stands fifth, and liver cancer stands third. More than 80% of these cases are found in the eastern Pacific and sub-Saharan African regions where tumor incidence is highest [5]. Despite the uncertainty surrounding malignancy caused by HBV, previous research has established that the HBV X (HBx) protein plays a significant role in HCC development. To bridge the gap between previous and present research information, the importance of HBx as a potential drug target for treating HCC was investigated [6].

Over 78,000 people die yearly from diseases of the liver that are both acute and chronic caused by the hepatitis B virus (HBV), and there are more than 255 million people infected chronically [7]. Cirrhosis and hepatocellular carcinoma are common complications associated with chronic hepatitis B in untreated adults. The two crucial antiviral therapies are nucleos(t)ide analogs (NAs) and pegylated interferon (IFN) α (PEG-IFN-α). A functional cure for HBV is rare despite the effectiveness of NAs. Hepatitis B is rarely eliminated, and drug resistance is a major concern during long-term treatment [8]. Despite the limited course of treatment and the possibility of maintaining a virologic response post drug withdrawal, PEG-IFN has not yet proved to be an effective treatment [9].

Numerous signaling pathways affected by the HBV X protein (HBx) influence cell invasion and proliferation. Aside from its role in viral replication and chromosomal instability, HBx plays a role in oncogenesis. DNA methylation, angiogenesis, oncogenesis, oxidative stress, and migration are all factors that it regulates [10].

## 2. Materials and Methods

### 2.1. Target Protein Accession

**Figure 1:**
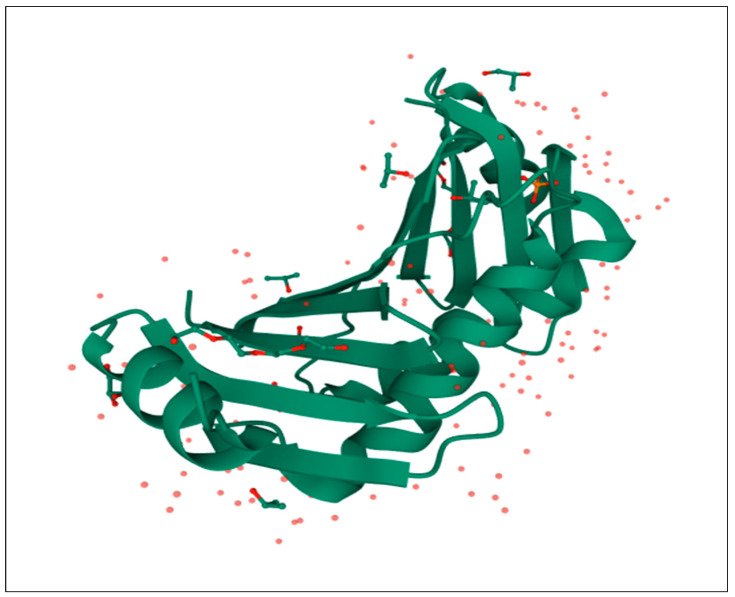
Structure of 3MSH protein.

The high-resolution crystal structure of the target, hepatitis B X-interacting protein, (1.51 Å) was taken from the RCSB Protein Data Bank (PDB ID-3MSH). The three-dimensional structure of protein HBx was obtained from RCSB PDB (Figure 1). The experimental data was obtained by X-ray crystallography.

### 2.2. Sequence Retrieval

The FASTA sequence of HBx protein with accession ID—3MSH_A of 99 amino acids was retrieved from NCBI.

>pdb|3MSH|A Chain A, hepatitis B virus X-interacting protein

MEATLEQHLEDTMKNPSIVGVLCTDSQGLNLGCRGTLSDEHAGVISVLAQQAAKLTSDPTDIPVVCLESD

NGNIMIQKHDGITVAVHKMASLEHHHHHH

### 2.3. Preparation of Ligands

From RCSB PDB, four unique ligands were identified, namely PG4, PO4, GOL, and IPA, from which PG4 was considered for further study since it is not commonly found in other proteins. A total of 20 ligands were selected using isomeric SMILES format from the PubChem database based on similar ligands in PDB and files were downloaded in 3D SDF format. The SMILES translator and structure file generator was used to convert the files into PDB format.2.4. for molecular docking analysis.

Docking is a computer-aided prediction of the size and conformation of drug and enzyme/protein seeking to find the best match between two molecules. Simply defined, docking is an in silico method that is used to predict a protein’s (enzyme) reaction with ligands.

Major steps involved in the docking process are:

Target selection > ligand selection and preparation > docking > evaluating docking results.

Large databases of potential drugs can be screened in silico to identify molecules with a high likelihood of binding to a target protein. Ligands are positioned correctly in a protein’s binding pocket during the docking process, and the affinity between the ligand and the protein is predicted.

The molecular docking procedure generates multiple ligand conformations and orientations that fit against the target and selects appropriate matches. The less the binding free energy of a complex, the more stable it is. To perform docking analysis, iGEMDOCK was used [11]. It uses an empirical scoring function and generic evolutionary method for the molecular docking process. This tool identifies pharmacological interactions visually, and virtual screening is performed through a graphical user interface. The screening process evaluates pharmacological interactions without using any of the known active compounds [12].

Compared to other docking simulation software, iGEMDOCK (version 2.1) displayed better overall results. GEMDOCK (Generic Evolutionary Method for Molecular DOCKing) is a tool for calculating the form and orientation of ligands in relation to the target protein. iGEMDOCK can be used both to prepare an interactive screening compound library and the target protein binding site [13]. A series of interaction profiles for protein-compound interactions are generated by iGEMDOCK, including electrostatic force (E), hydrogen-bonding (H), and van der Waal’s (V) interactions. As a final step, iGEMDOCK also allows individual screening compounds to be ranked and viewed according to their chemical activity and pharmacological interactions [12,14].

### 2.4. ADMET Studies

The selected hit molecules will be validated with ADME/T studies to identify potential lead molecules against the pathogenic organism. By using ADME/T tools, it is possible to predict pharmacokinetic parameters, such as the bioavailability, metabolic half-life, and permeability of the ligands during the drug design process [15]. Analyzing ADME during the initial discovery phase can dramatically reduce the fraction of clinical trials affected by pharmacokinetic failures [16].

Six physicochemical properties assessed for study were: lipophilicity, size, polarity, solubility, flexibility and saturation for the bioavailability radar. For a molecule to be considered drug-like, it has to be wholly within a physicochemical range on each axis which is depicted by the pink area in the radar graph [17].

Lipinski proposed ADMET properties called the “rule of five”. A compound can be evaluated for oral absorption by the rule of five, the oldest and most well-known of all the rules used to measure drug-likeness [18].

The Lipinski rule of five includes:

the molecular weight of molecule (MW) ≤ 500,
the octanol/water partition coefficient (iLOGP = A log P) ≤ 5,
the number of hydrogen bond donors (HBDs) ≤ 5,
the number of hydrogen bond acceptors (HBAs) ≤ 10.6, and,
the topological polar surface area (TPSA) < 40 Å^2^.

Apart from Lipinski’s rule, other rules that the compounds should adhere to are those of Ghose, Egan, Veber and Muegge. Each of these evaluate drug-like properties based on distinct parameters. A molecule can be orally bioactive/absorbable only if there is no violation of more than two of the rule of five conditions [19]. Some complex natural compounds that may not comply with this rule can be evaluated with several other drug-likeness rules equivalent to the rule of five [20].

### 2.5. Molecular Dynamics

Molecular dynamics simulation was performed using Schrödinger. This powerful computational tool can predict material properties, design drugs and model biomolecules, and much more [21]. MD simulation is performed after docking to optimize the final structures, analyze the stability of different complexes, and account for solvent effects as a final filter in silico to guide chemical synthesis for hit optimization [22].

It enables understand of structure and dynamics—analyzing the time-dependent behavior of a molecular system allows tracking of the motion of individual atoms at these scales [23]. The Schrödinger tool was used to analyze the parameters of MD trajectories, including: root mean square deviation (RMSD), root mean square fluctuation (RMSF), radius of gyration (RG), number of intermolecular hydrogen bonds, solvent accessible surface areas (SASA), and the B-factor [24].

## 3. Results and Discussion

### 3.1. Molecular Docking

Twenty ligands from PubChem were considered for the docking process. PDB format of the drug was chosen for preparing the binding site. The virtual screening procedure of iGEMDOCK consisted of four main steps which were: setting population size = 200, generations = 70, number of solutions = 2, and default setting = standard docking.

To evaluate binding affinities and to understand the possible interactions between ligands and proteins, molecular docking was performed. The energy contribution by van der Waal’s force, hydrogen bonding, and the electrostatic force is displayed in Table 1.

The top six ligands with least binding energy were: 2-[2-(2-ethoxyethoxy) ethoxy] ethanol (−59.0259), 2-[2-[2-(2-ethoxyethoxy) ethoxy] ethoxy] ethanol (−55.8216), 2-[2-[2-(2-propoxyethoxy) ethoxy] ethoxy] ethanol (−53.6121), 2-[2-[2-[2-(2-hydroxyethoxy) ethoxy] ethoxy] ethoxy] ethanol (−52.5801), 2-[2-[2-(2-methoxyethoxy) ethoxy] ethoxy] ethanol (−52.1195), and 2-[2-(2-propoxyethoxy) ethoxy] ethanol (−51.3424). The best molecule showing the highest binding energy, 2-[2-(2-ethoxyethoxy) ethoxy] ethanol, was the most effective inhibitor.

**Figure 2:**
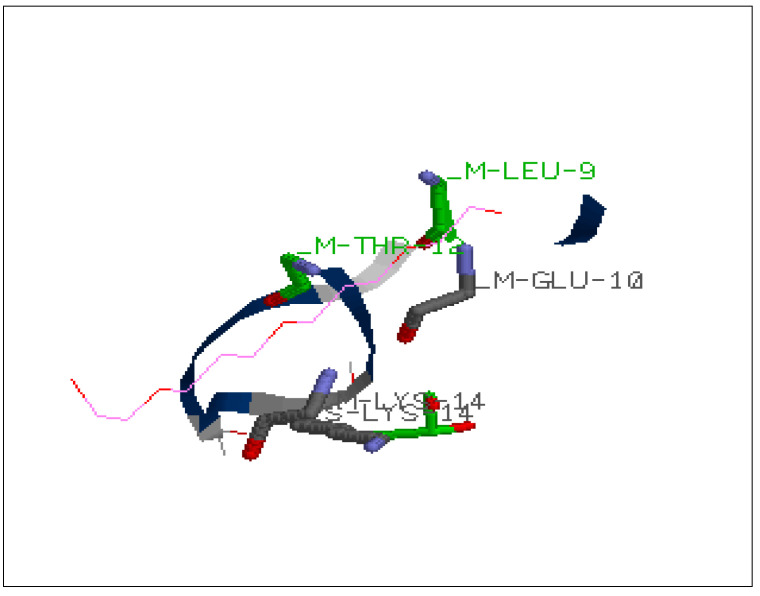
Interaction analysis viewed in RasMol for CID_8190 (RasWin Molecular Graphics).

Amino acids contributing to the binding of the compound can be viewed in the interaction analysis depicted in Table 2. The amino acids which were involved in interaction with the protein were 9 (leucine), 10 (glutamine), 12 (threonine), 15 (asparagine) as depicted in Figure 2.

### 3.2. ADMET Studies

The selected molecules had an acceptable oral toxicity (LD50), which means they would not elicit any untoward adverse effects in low concentrations. As a result of the analysis of all properties, the molecules in question were determined to be non-toxic, ensuring their safety. The predicted toxicity properties of the molecules, along with their prediction probability, bioavailability and drug-likeness, are shown in Table 3, Table 4 and Table 5.

Considering CID_8190, after docking results, it was observed that the compound followed the rules of Lipinski, Ghose, Veber, Egan and Muegge with a bioavailability score of 0.55.

**Figure 3:**
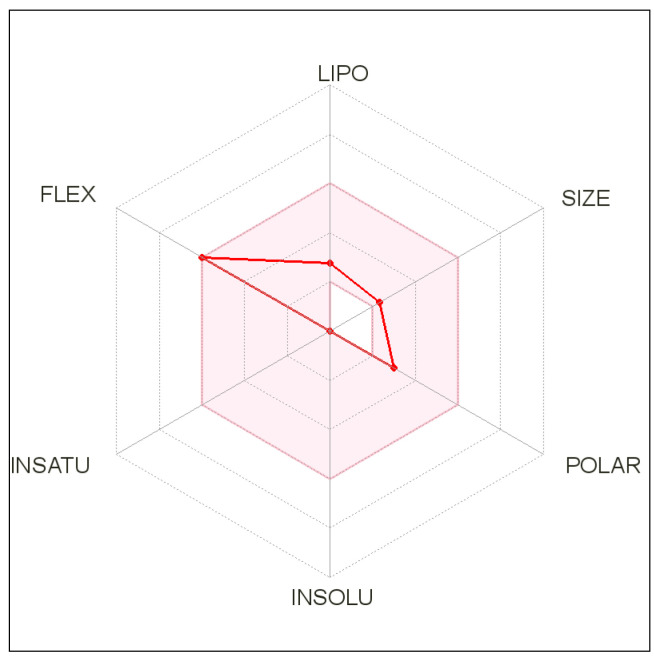
Bioavailability radar for CID_8190.

The bioavailability radar has six axes which consist of six essential properties for oral bioavailability. The optimum values are depicted in the pink region. The red line of the compound under consideration was completely included in the pink area. This shows that the criteria of flexibility, lipophilicity, size and polar nature were fulfilled (Figure 3).

According to the ADME and drug-like properties of the molecules shown above, the molecules are highly bioavailable in the gastrointestinal tract, but not permeable through the blood-brain barrier (BBB).

The bioavailability radar considers six physicochemical properties of a drug to determine the molecule’s drug-likeness: saturation, polarity, flexibility, size, lipophilicity, and solubility [25].

**Figure 4:**
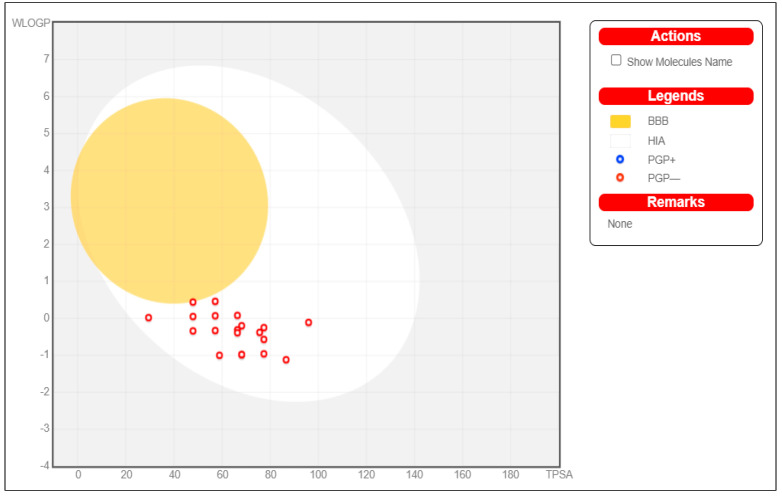
Molecules represented as a boiled egg graph.

The molecules were shown to be bioavailable orally, of low toxicity, and to have a good absorption rate (Figure 4).

### 3.3. MD Simulation

All the protein frames were initially aligned on the reference frame backbone, and the calculation of RMSD was based on Cα or side chain. Visualizing the RMSD of protein provides detailed information concerning structural conformations during the simulation. This parameter indicates simulation equilibration and its fluctuation around a thermal mean. Changes of the order of 1–3 Å are acceptable. If the protein undergoes a much more significant conformational change than 3 Å, it indicates that a large conformational change occurs during simulation. RMSD values should stabilize at around a fixed value or converge during simulation. An insight into the ligand’s stability relative to the binding pocket of the protein is provided by ligand RMSD.

**Figure 5:**
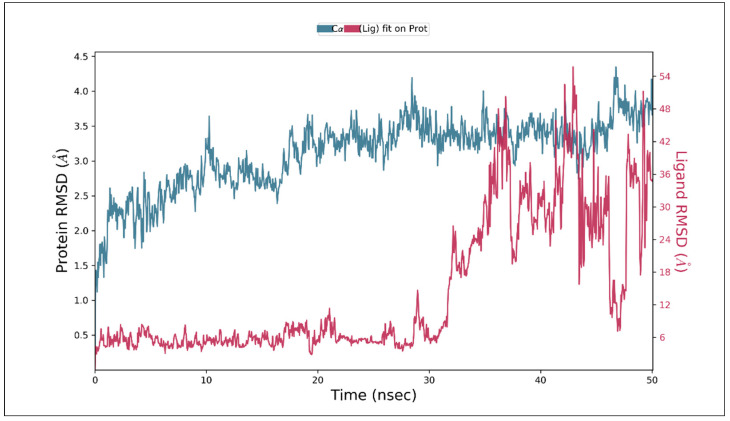
Protein-ligand RMSD graph of MD simulation trajectory.

The graph in Figure 5 shows the protein RMSD evolution (indicated on the left *Y*-axis) and ligand RMSD (indicated on the right *Y*-axis). The plot shows that the compound hepatitis B X-interacting protein (PDB ID 3MSH) complex showed stabilization soon after beginning the simulation, i.e., 10 ns. Considering ligand RMSD, the fluctuation was observed after 30 ns of the trajectory curve. Throughout the simulation of 50 ns, no noteworthy conformational changes occurred in the protein structure. Variations were in the range of 1–3 Å, which can be considered to be non-significant.

**Figure 6:**
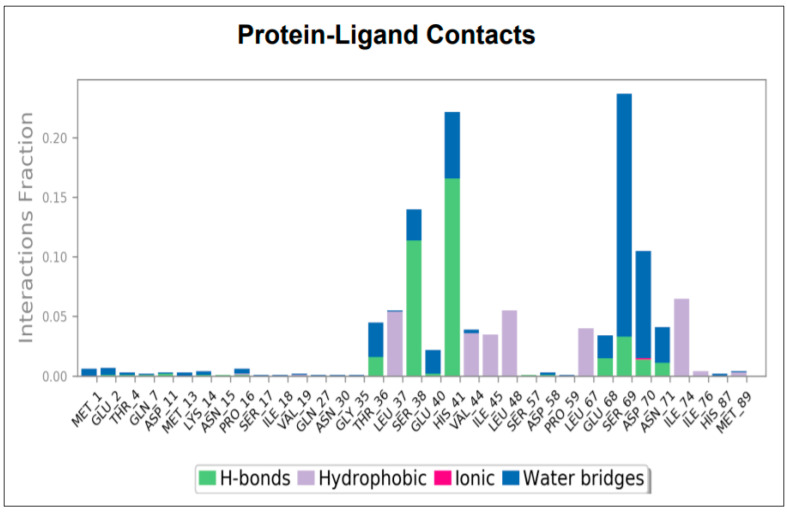
Protein-ligand contacts after the simulation of molecular dynamics.

Protein-ligand interactions are divided into four types: hydrogen bonds, hydrophobic and ionic interactions, and water bridges. The graph displays the compound contacts studied during the 50 ns trajectory in bar chart format (Figure 6). The bar graph depicts hydrogen bonds, hydrophobic contacts, and water bridges visualized throughout the simulation period. The significant hydrogen bonds observed in the initial docked pose of the compound (Thr 36, Ser 38, Glu 68, His 41, Asp 70, Ser 69, Asn 71) did not change during the simulation. Hydrogen bonding with His 41 remained for more than 20% of the simulation time. Hydrophobic interaction was observed with residues Leu 37, Val 44, Ile 45, Leu 48, Leu 67, Ile 74, and Ile 76. Water bridged interactions were noticed with residues Thr 36, Ser 38, Glu 40, Glu 68, Ser 69, Asp 70, and Asn 71. Hence, the four important residues identified were: Ser 38, His 41, and Ser 69.

## 4. Conclusions

With the advancement of technology, computer-aided drug design (CADD) has paved the way for lead identification and optimization in research and development. Using in silico tools, it is easier and more effective to limit the required number of molecules for further analysis by experiments. The study identified twenty compounds from which triethylene glycol monoethyl ether derivative was chosen based on docking score, binding energies, suitable ADMET, and simulation results. In conclusion, this research has highlighted the relevance of this compound as a potential treatment lead for hepatitis B, which could be used for developing more potent anti-HBV drugs.

## Figures and Tables

**Table 1:** Docking results for the selected molecules (kcal/mol).

S. No.	Ligand	Total Energy	VDW	H Bond	Elec	AverConPair
1	8190-1.pdb	−59.0259	−37.4125	−21.6135	0	18.4667
2	79734-0.pdb	−55.8216	−40.0817	−15.7399	0	18.4667
3	12527511-1.pdb	−53.6121	−38.0934	−15.5187	0	18.4375
4	62551-1.pdb	−52.5801	−36.7199	−15.8603	0	18.4375
5	90263-0.pdb	−52.1195	−38.2955	−13.824	0	18.5
6	11355992-0.pdb	−51.3424	−36.5669	−14.7755	0	18.4615
7	154263124-0.pdb	−49.9133	−31.9133	−18	0	18.4667
8	140933500-1.pdb	−46.1511	−32.1511	−14	0	18.4667
9	140264802-0.pdb	−45.7681	−41.1789	−4.58916	0	18.6
10	90255-1.pdb	−44.617	−28.0377	−16.5793	0	18.4706
11	153406521-0.pdb	−44.5652	−44.5652	0	0	18.5294
12	149434283-1.pdb	−43.7893	−30.4657	−13.3236	0	18.5
13	8200-1.pdb	−43.0239	−29.5751	−13.4488	0	18.5385
14	8178-0.pdb	−42.8289	−34.5198	−8.30908	0	19
15	45281241-1.pdb	-42.785	−36.6431	−6.14197	0	19
16	78058-1.pdb	−41.9812	−41.9812	0	0	18.5
17	150190459-1.pdb	−40.4711	−40.4711	0	0	18.5556
18	8172-0.pdb	−40.1369	−31.7043	−8.4326	0	19.5
19	13811968-0.pdb	−39.4177	−32.4177	−7	0	18.4706
20	8076-1.pdb	−30.5437	−23.5437	−7	0	23.5

**Table 2:** Interaction analysis results for the selected molecules.

S. No.	Compound	Energy	H-M-LEU-9	H-M-GLU-10	H-M-THR-12	H-S-THR-12	H-M-ASN-15	H-S-ASN-15
1	8190-1.pdb	−59	−6.73215	−6.99712	−0.884197	0	−3.5	−3.5
2	79734-0.pdb	−55.8	−3.5	0	−9.3506	−2.5	−0.38927	0
3	12527511-1.pdb	−53.6	−3.47972	0	−8.62373	−2.5	−0.915265	0
4	62551-1.pdb	−52.6	−3.5	0	−8.27415	−2.5	−1.5861	0
5	90263-0.pdb	−52.1	−3.5	−3.5	−3.5	0	−3.32398	0
6	11355992-0.pdb	−51.3	−3.5	−3.09354	−7	0	−1.18197	0
7	154263124-0.pdb	−49.9	0	0	−10.5	−7.5	0	0
8	140933500-1.pdb	−46.2	−3.5	0	−7	0	−3.5	0
9	140264802-0.pdb	−45.8	0	0	0	0	0	0
10	90255-1.pdb	−44.6	−4.86515	0	−10.5	−1.21414	0	0
11	153406521-0.pdb	−44.6	0	0	0	0	0	0
12	149434283-1.pdb	−43.8	−3.13025	0	−7	0	−3.19338	0
13	8200-1.pdb	−43	−3.46947	0	−7	0	−2.97934	0
14	8178-0.pdb	−42.8	0	0	−3.5	0	−1.47745	−3.33163
15	45281241-1.pdb	−42.8	0	0	−3.5	0	−0.966519	−1.67545
16	78058-1.pdb	−42	0	0	0	0	0	0
17	150190459-1.pdb	−40.5	0	0	0	0	0	0
18	8172-0.pdb	−40.1	0	0	−3.5	0	−1.4326	−3.5
19	13811968-0.pdb	−39.4	0	0	0	0	0	0
20	8076-1.pdb	−30.5	0	0	−3.5	0	−3.5	0

**Table 3:** Properties of selected molecules related to Lipinski’s Rule.

S. No.	CID	Mol Wt	H Bond Acceptors	H Bond Donors	TPSA	iLOGP	Lipinski Violations
1	CID_8076	90.12	2	1	29.46	1.66	0
2	CID_8172	150.17	4	2	58.92	1.57	0
3	CID_8178	164.2	4	1	47.92	2.21	0
4	CID_8190	178.23	4	1	47.92	2.41	0
5	CID_8200	194.23	5	2	68.15	2.38	0
6	CID_62551	238.28	6	2	77.38	2.9	0
7	CID_78058	266.33	6	1	66.38	3.65	0
8	CID_79734	222.28	5	1	57.15	3.01	0
9	CID_90255	252.3	6	1	66.38	3.19	0
10	CID_90263	208.25	5	1	57.15	2.84	0
11	CID_11355992	192.25	4	1	47.92	2.74	0
12	CID_12527511	236.31	5	1	57.15	3.34	0
13	CID_13811968	252.3	6	2	77.38	2.84	0
14	CID_45281241	222.28	5	2	68.15	2.36	0
15	CID_140264802	226.22	7	2	86.61	2.65	0
16	CID_140933500	194.23	5	2	68.15	2.38	0
17	CID_149434283	226.29	5	1	95.95	0	0
18	CID_150190459	268.3	7	1	75.61	3.34	0
19	CID_153406521	256.27	7	2	77.38	2.75	0
20	CID_154263124	224.25	6	1	66.38	2.95	0

**Table 4:** Computed pharmacokinetic parameters of the selected molecules.

S. No.	CID	GI Absorption	BBB Permeant	Pgp Substrate	CYP1A2 Inhibitor	CYP2C19 Inhibitor	CYP2C9 Inhibitor	CYP2D6 Inhibitor	log Kp (cm/s)
1	CID_8076	High	No	No	No	No	No	No	−7.08
2	CID_8172	High	No	No	No	No	No	No	−8.34
3	CID_8178	High	No	No	No	No	No	No	−8.04
4	CID_8190	High	No	No	No	No	No	No	−7.88
5	CID_8200	High	No	No	No	No	No	No	−8.61
6	CID_62551	High	No	No	No	No	No	No	−8.98
7	CID_78058	High	No	No	No	No	No	No	−8.62
8	CID_79734	High	No	No	No	No	No	No	−8.25
9	CID_90255	High	No	No	No	No	No	No	−8.69
10	CID_90263	High	No	No	No	No	No	No	−8.32
11	CID_11355992	High	No	No	No	No	No	No	−7.43
12	CID_12527511	High	No	No	No	No	No	No	−7.85
13	CID_13811968	High	No	No	No	No	No	No	−8.82
14	CID_45281241	High	No	No	No	No	No	No	−8.27
15	CID_140264802	High	No	No	No	No	No	No	−8.74
16	CID_140933500	High	No	No	No	No	No	No	−8.61
17	CID_149434283	High	No	No	No	No	No	No	−8.09
18	CID_150190459	High	No	No	No	No	No	No	−8.75
19	CID_153406521	High	No	No	No	No	No	No	−8.73
20	CID_154263124	High	No	No	No	No	No	No	−8.38

**Table 5:** Computed drug-likeness properties.

S. No.	CID	Lipinski	Ghose	Veber	Egan	Muegge	Bioavailability Score
1	CID_8076	0	3	0	0	2	0.55
2	CID_8172	0	3	0	0	1	0.55
3	CID_8178	0	0	0	0	1	0.55
4	CID_8190	0	0	0	0	1	0.55
5	CID_8200	0	1	0	0	1	0.55
6	CID_62551	0	1	1	0	0	0.55
7	CID_78058	0	0	1	0	0	0.55
8	CID_79734	0	0	1	0	0	0.55
9	CID_90255	0	0	1	0	0	0.55
10	CID_90263	0	0	1	0	0	0.55
11	CID_11355992	0	0	0	0	1	0.55
12	CID_12527511	0	0	1	0	0	0.55
13	CID_13811968	0	1	1	0	0	0.55
14	CID_45281241	0	0	1	0	0	0.55
15	CID_140264802	0	1	1	0	0	0.55
16	CID_140933500	0	1	0	0	1	0.55
17	CID_149434283	0	0	1	0	0	0.55
18	CID_150190459	0	0	1	0	0	0.55
19	CID_153406521	0	0	1	0	0	0.55
20	CID_154263124	0	0	1	0	0	0.55

## Data Availability

All the data has been presented in this article.
